# Pneumocystis jirovecii Pneumonia and HIV-Associated Nephropathy in Acute HIV Infection

**DOI:** 10.7759/cureus.69189

**Published:** 2024-09-11

**Authors:** Sina Hedayatpour, Alla Albonijim, Juan Avila

**Affiliations:** 1 Internal Medicine, Methodist Health System, Dallas, USA; 2 Internal Medicine, Methodist Dallas Medical Center, Dallas, USA

**Keywords:** hiv aids, hiv associated nephropathy (hivan), internal medicine, pjp in hiv, pneumocystis jiroveci pneumonia, pulmonology

## Abstract

HIV is a retrovirus that affects the body’s immune system, primarily dendritic cells, macrophages, and CD4^+^ T cells. As a result, several opportunistic infections are associated with HIV infection, including *Pneumocystis jirovecii *pneumonia (PJP), *Toxoplasma gondii *(toxoplasmosis), *Cryptococcus *(cryptococcosis), and *Mycobacterium avium *complex (MAC) infection. HIV is also associated with acute kidney injury and chronic kidney disease. The classic kidney disease related to HIV is HIV-associated nephropathy (HIVAN). HIVAN pathogenesis is linked to glomerular and renal tubular epithelial cell infection. With the advances in antiretroviral therapy, patients with HIV can live an expected lifespan without progression to AIDS and AIDS-related complications. Therefore, it is important for clinicians to recognize new-onset HIV and the complications associated with HIV. Our patient, a 32-year-old male, presented with two weeks of productive cough and one week of diarrhea. He was diagnosed with HIV and PJP based on HIV antigen/antibody testing and a sputum PJP PCR assay, respectively. The patient also had an acute kidney injury with likely underlying kidney disease suspicious of HIVAN. The patient underwent treatment for PJP and was discharged in stable condition with PJP prophylaxis.

## Introduction

HIV is associated with several opportunistic infections, including *Pneumocystis jirovecii* pneumonia (PJP), *Toxoplasma gondii *(toxoplasmosis), and *Mycobacterium avium* complex (MAC). Opportunistic infections can occur at any stage of HIV infection but usually occur when the CD4^+^ T cell count is <200 cells/μl [1]. PJP is one of the most frequent AIDS-associated illnesses in North America and Europe [2]; however, the incidence of PJP has decreased since the advent of anti-retroviral therapy (ART). For example, San Francisco General Hospital had noted approximately 250 cases of HIV-associated PJP per year prior to combination ART. After the ART era, the number of cases reported decreased to 20-30 cases per year [2]. Most cases occur in patients who are not aware of their HIV diagnosis, do not receive ART or do not receive PJP prophylaxis [2]. Thus, the diagnosis and treatment of HIV and AIDS-associated illnesses are important for today’s physicians to recognize, especially in patients who present with nonspecific symptoms.

Much like opportunistic infections, end organ damage can be present in HIV patients late in the course of illness. HIV patients presenting with chronic kidney disease (CKD) may also have underlying HIV-associated nephropathy (HIVAN). This is often in the setting of proteinuria and ultrasound findings of enlarged, echogenic kidneys. HIVAN pathogenesis is linked to glomerular and renal tubular epithelial cell infection. In renal biopsy, this may be seen as a collapsing form of focal segmental glomerulosclerosis [3]. Definitive diagnosis requires a kidney biopsy, as more than 30% of patients with suspected HIVAN will have an alternate diagnosis based on histology [3]. Our clinical case highlights a patient with newly diagnosed AIDS, PJP pneumonia (confirmed via sputum PCR), and underlying CKD concerning HIVAN.

## Case presentation

A 32-year-old male with no known past medical history presented with a two-week history of progressively worsening cough productive of white/clear sputum and a one-week history of diarrhea. A review of systems was notable for a non-pruritic facial rash, which initially presented one month prior to admission. The patient otherwise denied subjective fevers, dyspnea, chest pain, nausea, vomiting, abdominal pain, or dysuria. He denied having any sick contacts, recent travel, or hospitalizations.

On physical examination, vital signs were notable for tachycardia (110 beats per minute). He was alert and oriented to person, place, time, and situation with dry oral mucosa. A facial rash was present, which was flat and erythematous across the bridge of the nose and cheeks, sparing the nasolabial folds. Coarse breath sounds were noted throughout all lung fields. Pertinent admission laboratory data included normocytic anemia with a hemoglobin of 8.7 mg/dL and mean corpuscular volume of 90.2 fL, an elevated blood urea nitrogen level of 27 mg/dL, an elevated creatinine level of 2.76 mg/dL, proteinuria of 200 mg/dL on urinalysis, elevated inflammatory markers (i.e., ferritin = 1780 ng/mL, erythrocyte sedimentation rate = 81 mm/hr, C3 = 169 mg/dL, and C4 = 66 mg/dL) and mild hyperphosphatemia and hypoalbuminemia. Iron studies showed findings consistent with anemia of chronic disease. A chest X-ray showed reticular opacities over the upper lungs bilaterally, left more than right. A renal ultrasound showed echogenic kidneys bilaterally, consistent with medical renal disease.

**Figure 1:**
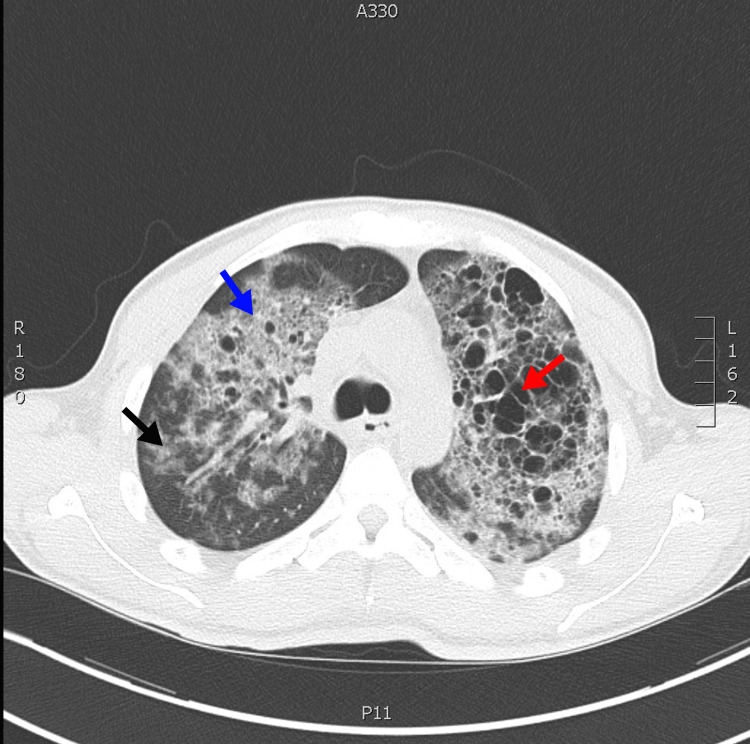
Computed tomography of the chest showed extensive ground glass opacities (black arrow) with consolidative and nodular features (blue arrow) present in both lungs with areas of cystic proliferation (red arrow). Upper lobe predominance noted

Given his history of facial rash, anemia of chronic disease, proteinuria, elevated inflammatory markers, and abnormal ultrasound, there was a concern for underlying intrinsic renal pathology. His renal function improved with IV fluids. Inpatient nephrology was consulted for consideration of a kidney biopsy, which the patient ultimately declined. After obtaining consent, HIV testing was obtained given his young age, diarrhea, facial rash, productive cough, and chest imaging findings consistent with suspected pulmonary infection. He was positive for HIV-1 with a viral load >900K copies/mL and a CD4^+^ T cell count of 22 cells/mm^3^ (5.6%). He was thus diagnosed with AIDS and infectious disease was consulted for additional assistance in patient management. During his hospital course, he became febrile, dyspneic, and hypoxic requiring supplemental oxygen via nasal cannula. The patient’s (1,3) β-d-glucan level was >500 pg/mL (suggestive of a *P. jirovecii *infection), and further imaging with computed tomography of the chest showed extensive ground glass opacities, which was highly suspicious for PJP (Figure 1). He was subsequently started on oral double-strength trimethoprim-sulfamethoxazole (TMP-SMX) three times daily with a planned duration of 21 days of treatment. The patient was unwilling to undergo arterial blood gas testing for alveolar-arterial (A-a) gradient evaluation; however, in the setting of persistent O_2_ requirements and high clinical suspicion for PJP, steroid initiation was favored with a prednisone taper. The patient was ultimately diagnosed with PJP based on the results from a sputum PCR assay.

While on TMP-SMX, the patient developed hyperkalemia and was eventually transitioned to primaquine and clindamycin for further treatment. In addition to his PJP-directed antibiotic regimen and steroid taper, the patient was discharged on dapsone for PJP prophylaxis. He was referred to outpatient nephrology and infectious disease clinics for surveillance of his kidney function and eventual initiation of ART.

## Discussion

This case report illustrates the presentation of acute kidney injury with likely underlying kidney disease suspicious of HIVAN in a patient with *P. jirovecii* infection. HIVAN is classically associated with CKD and end-stage renal disease (ESRD) in HIV-1 seropositive patients, and it usually presents late in the course of HIV infection [4]. Patients with HIVAN are typically present with nephrotic syndromes consisting of nephrotic-range proteinuria (>3.5 g/day), azotemia, hypoalbuminemia, and hyperlipidemia. The diagnosis is usually made when patients reach the advanced kidney failure stage with severe proteinuria but no notable peripheral edema or hematuria. The renal prognosis for HIVAN is very poor. In untreated patients, HIVAN causes a rapid renal function deterioration that can result in ESRD within two to four months after diagnosis [4,5]. While laboratory findings are largely nonspecific, urinalysis will commonly reveal proteinuria. Furthermore, renal ultrasound imaging may show enlarged bilateral echogenic kidneys but usually reveals normal-sized kidneys in the advanced stages of the disease. The only reliable method of diagnosing HIVAN is renal biopsy regardless if the patient has significant proteinuria with HIV infection. HIVAN pathogenesis involves the abnormal response of podocytes to HIV-1 in renal parenchymal cells, which leads to dysregulation of the cell cycle of glomerular and tubular epithelial cells with increased proliferation, apoptosis, cellular dedifferentiation, and altered cellular polarity [6,7].

Treatment of HIVAN involves mostly ART, angiotensin-converting enzyme inhibitors (ACEi), and steroids, with the goal of slowing HIV-1 replication and the progression of kidney disease. ACEi in combination with highly active ART (HAART) significantly decreases proteinuria and the rate of decline in the glomerular filtration rate as well as lowers the frequency of ESRD [8]. ACEi and HAART are effective in preventing and reversing HIVAN-induced renal failure in some cases. For those who do not respond to the above-mentioned treatments, a short course of a corticosteroid may result in long-standing disease remission [5]. Low-dose prednisone reduces general immune activation, which is the key factor for HIV disease progression. It also has a mortality benefit [9]. Non-pharmacologic treatment methods include dialysis and kidney transplantation. The initiation of dialysis improves survival in patients with an earlier stage of HIV infection, younger age, and higher CD4^+^ T cell counts as well as those on HAART therapy [4]. In kidney transplant patients with HIVAN, one- and two-year graft survival is comparable to other high-risk populations receiving kidney transplants. Also, one- and two-year patient survival in HIVAN transplant patients is higher than those on dialysis. In the short term, immunosuppression has not been shown to adversely affect HIV patients on HAART [10]. Transplant should only be considered in a carefully selected group of patients.

*P. jirovecii* is an opportunistic fungal pathogen that can cause severe pneumonia (i.e., PJP) in immunocompromised hosts. First-line therapy for PJP prophylaxis and treatment remains TMP-SMX, as randomized control trials have found this antibiotic to have the best overall efficacy. Alternate treatment is recommended when there are notable side effects (i.e., hyperkalemia), intolerance, allergy, or treatment failure. Adjunctive therapy to augment standard therapy include dapsone, pentamidine, atovaquone, clindamycin and primaquine [11]. Patients with CD4^+^ T cell counts <200 cells/mm^3^ and CD4^+^ T cell percentages <14% should receive chemoprophylaxis against PJP with one double-strength TMP-SMX tablet daily [12]. This will also provide cross protection against toxoplasmosis and many respiratory bacterial infections [13]. If TMP-SMX is discontinued due to a mild adverse reaction, re-institution can be considered when the reaction has resolved. TMP-SMX should be permanently discontinued in patients with life-threatening adverse reactions including Stevens-Johnson syndrome or toxic epidermal necrolysis. Alternative prophylactic regimens include dapsone; dapsone plus pyrimethamine and leucovorin; aerosolized pentamidine with a Respirgard II nebulizer; and atovaquone [13]. Prophylaxis can be discontinued in patients whose CD4+ T cell counts increase from <200 cells/mm^3^ to >200 cells/mm^3 ^for >3 months while on HAART [14]. For mild to moderate PJP, the preferred treatment is double-strength TMP-SMX three times daily for 21 days. For moderate to severe PJP, the preferred therapy is TMP-SMX based on weight (TMP 15 to 20 mg and SMX 75 to 100 mg)/kg/day IV every six to eight hours. Adjunctive corticosteroids with prednisone taper for 21 days are considered if the following criteria is met: PaO_2 _< 70 mmHg at room air or an A-a gradient ≥ 35 mmHg [15] (Table 1).

**Table 1:** Treatment of PJP PJP: *Pneumocystis jiroveci* pneumonia; TMP: trimethoprim; SMX: sulfamethoxazole; DS: double strength; PO: orally; IV: intravenously Reference: [15]

Mild to moderate PJP pneumonia treatment: total duration of 21 days	
Preferred therapy	TMP-SMX: (TMP 15-20 mg/kg/day and SMX 75-100 mg/kg/day) PO (three divided doses) or
	TMP-SMX 2 DS tablets PO three times daily
Alternative therapy	Dapsone 100 mg PO daily plus TMP 15 mg/kg/day PO (three divided doses) or
	Primaquine 30 mg (base) PO daily plus Clindamycin PO (450 mg every six hours or 600 mg every eight hours) or
	Atovaquone 750 mg PO twice daily with food
Moderate to severe PJP pneumonia treatment: total duration of 21 days	
Preferred therapy	TMP-SMX: ((TMP 15-20 mg and SMX 75-100 mg)/kg/day IV given every six or eight hours)
	May switch to PO formulations after clinical improvement
Alternative therapy	Pentamidine 4 mg/kg IV once daily infused over ≥60 minutes
	May reduce the dose to pentamidine 3 mg/kg IV once daily the event of toxicities or
	Primaquine 30 mg (base) PO once daily plus (Clindamycin (IV 600 mg every six hours or 900 mg every eight hours) or
	(PO 450 mg every six hours or 600 mg every eight hours))
Adjunctive corticosteroids	
For moderate to severe PJP based on the following criteria:	
PaO_2_ <70 mmHg at room air or	
Alveolar-arterial DO_2_ gradient ≥35 mmHg	
Prednisone (beginning as soon as possible and within 72 hours of PCP therapy)	
	Days 1-5: 40 mg PO twice daily
	Days 6-10: 40 mg PO daily
	Days 11-21: 20 mg PO daily
IV methylprednisolone can be given as 75% of prednisone dose	

## Conclusions

The early and accurate diagnosis of the complications of HIV remains to be a clinical imperative for today’s physicians. This is especially true in the context of AIDS, as the course is often indolent. The advent of ART has caused a decline in the incidence of this disease and may be overlooked as a differential diagnosis. This may be considerably harmful, as delayed diagnosis and treatment are associated with higher mortality. Furthermore, it is important for clinicians to recognize early diagnosis of HIV and initiate ART therapy to prevent morbidity and mortality. This case highlights the complications of untreated or unknown HIV status, such as PJP and HIVAN, which span multiple organ systems along with the diagnostic and treatment pathways. In addition, this case suggests future areas of research with regard to risk factors for these complications as well as non-invasive tools for the diagnosis of these conditions.
